# Spatial Complementarity and the Coexistence of Species

**DOI:** 10.1371/journal.pone.0114979

**Published:** 2014-12-22

**Authors:** Jorge Velázquez, Juan P. Garrahan, Markus P. Eichhorn

**Affiliations:** 1 School of Physics & Astronomy, The University of Nottingham, University Park, Nottingham, NG7 2RD, United Kingdom; 2 Facultad de Ciencias Físico Matemáticas, Universidad Autónoma de Puebla, 72001, Puebla, Pue., México; 3 School of Life Sciences, The University of Nottingham, University Park, Nottingham, NG7 2RD, United Kingdom; University of Saskatchewan, Canada

## Abstract

Coexistence of apparently similar species remains an enduring paradox in ecology. Spatial structure has been predicted to enable coexistence even when population-level models predict competitive exclusion if it causes each species to limit its own population more than that of its competitor. Nevertheless, existing hypotheses conflict with regard to whether clustering favours or precludes coexistence. The spatial segregation hypothesis predicts that in clustered populations the frequency of intra-specific interactions will be increased, causing each species to be self-limiting. Alternatively, individuals of the same species might compete over greater distances, known as heteromyopia, breaking down clusters and opening space for a second species to invade. In this study we create an individual-based model in homogeneous two-dimensional space for two putative sessile species differing only in their demographic rates and the range and strength of their competitive interactions. We fully characterise the parameter space within which coexistence occurs beyond population-level predictions, thereby revealing a region of coexistence generated by a previously-unrecognised process which we term the triadic mechanism. Here coexistence occurs due to the ability of a second generation of offspring of the rarer species to escape competition from their ancestors. We diagnose the conditions under which each of three spatial coexistence mechanisms operates and their characteristic spatial signatures. Deriving insights from a novel metric — ecological pressure — we demonstrate that coexistence is not solely determined by features of the numerically-dominant species. This results in a common framework for predicting, given any pair of species and knowledge of the relevant parameters, whether they will coexist, the mechanism by which they will do so, and the resultant spatial pattern of the community. Spatial coexistence arises from complementary combinations of traits in each species rather than solely through self-limitation.

## Introduction

It is a common maxim in ecology that in order for two species to coexist, each must limit the growth of its own population more than that of the other [1]. This prediction can be intuitively derived from an expectation that individuals of the same species will compete more strongly for identical resources. Such niche-based models of coexistence have substantial empirical and theoretical support [2]. Nevertheless, numerous examples exist where an apparently weaker species persists alongside a stronger competitor [3].

Competitive interactions among individuals occur over limited distances. For sessile organisms, such as plants or corals, the degree of competition experienced by any individual is influenced principally by others in its immediate neighbourhood [4], [5]. Furthermore, restricted dispersal makes it inevitable that populations and communities will have spatial structure [6]. Traditional ecological studies have tended to focus on the characteristics of entire populations, such as average sizes, growth rates, or density, and it is only in recent times that the importance of spatial patterning of individuals has been fully appreciated. When localised interactions outweigh population-level effects, levels of competition averaged across individuals become uninformative. Spatial organisation can therefore have major implications for the emergent properties of natural systems, including the coexistence of species [7].

We define spatial coexistence as occurring when the spatial structure of a community permits multiple species to persist indefinitely even when this would not be possible were all individuals to experience average environmental conditions [8]. There are many means by which this can occur, including trade-offs among species in rates of colonisation, competition and longevity [9], or through variation in environmental quality in space or time [10], [11]. In this study we seek the minimum conditions for two species to coexist in a uniform environment without the need for strict trade-offs.

Spatial organisation can either promote or preclude coexistence, though the former is expected when it causes competition within species to exceed that between. There are two means by which this might occur. The first is frequency-based: aggregation within species increases the frequency of intra-specific interactions relative to inter-specific, termed the spatial segregation hypothesis [12]. Multiple experimental studies have demonstrated that clustering of species enables coexistence, especially of weaker competitors [13]–[17]. The second is distance-based: the scale over which individuals of the same species interact might be greater than between species, a process known as heteromyopia [18]. Individuals of the dominant species become spread out, creating interstices in their spatial pattern which can be colonised by another species. This implies that it is the breakdown of aggregations which promotes coexistence. A debate has therefore developed surrounding the question of whether aggregation favours or impedes coexistence [18]–[20].

A common feature of most studies to date is that the problem posed is invariably how an inferior competitor is able to persist. In contrast, we believe that coexistence should be seen as a two-sided process; understanding the structure of a mixed community requires us to integrate the complementary forces driving the population dynamics of all participants.

In this study we generate a novel individual-based model for two-species competition in a uniform environment in which our species can differ in their demographic rates (birth, death, reproduction) and the intensity and range of competition. We explore the parameter space representing a wide range of potential species and show that the spatial structure of communities allows coexistence to occur even when population-level predictions suggest it to be impossible. We thereby reconcile existing theories within a common framework and reveal a previously unrecognised mechanism through which spatial structure enables the coexistence of similar species. Through this we demonstrate that spatial coexistence depends on complementary traits rather than self-limitation.

## Materials and Methods

### Simulation model

Our study is based upon a stochastic individual-based model (IBM) representing a notional community in which two sessile species with overlapping generations occupy a uniform environment. The code can be accessed as a git repository hosted at https://github.com/jorgevc/IBM-ecology-simulator.git. Individuals occur at sites determined by a two-dimensional grid (*x*, *y* co-ordinates). When reproducing it is assumed that individuals are limited in their ability to disperse offspring. Similarly, competition among individuals for resources only takes place within a fixed radius. The spatial patterns that arise are therefore a direct consequence of the dispersal of individuals and their interactions rather than any external driver. The parameters of the model are defined in Table 1. To develop the system we used standard procedures employed in simulations of statistical mechanics [21].

**Table 1:** Parameter definitions from the stochastic individual-based model used to develop the simulations.

Parameter	Definition
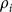	Density of species *i* measured as proportion of occupied sites
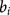	Birth rate of species *i*
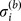	Dispersal radius 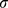 of new offspring of species *i*
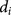	Intrinsic death rate of species *i*
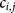	Competition rate by which species *j* kills species *i*
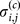	Competition radius within which species *j* can kill species *i*

The arena was a two-dimensional square lattice of 150 units, giving a total 22,500 sites, with periodic boundaries forming a torus to prevent inward propagation of edge effects. Birth and death events took place in continuous time. The starting density of each species in the lattice was 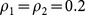 (the final output in the stationary state is not affected by this value). A relatively high starting density was chosen since invasibility was not used as the coexistence criterion within this study; an assessment of the implications of this distinction is reserved for the Discussion.

Initial individuals were distributed according to an homogeneous Poisson process (complete spatial randomness). In order to compensate for stochastic fluctuations due to the finite size of the system we present results based on ensemble averages. Each ensemble is composed of 20 realisations. The averages therefore have fluctuations equivalent to those of a single simulation performed on a lattice of 22,500×20 = 450,000 sites. The use of discrete space in our simulations is an optimisation to increase computational speed; results would not differ for ‘continuous’ space, which in modelling terms effectively means a lattice of higher resolution.

The frequency with which an individual belonging to species *i* makes an attempt to produce an offspring is given by the birth rate 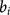. The site occupied by the new offspring is chosen with equal probability among all sites that are closer or equal to the distance 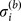 from its parent. If the randomly chosen site is already occupied then production of an offspring is prevented. Death of individuals can occur due to both intrinsic and extrinsic causes and therefore requires more than one parameter. The intrinsic death rate of species *i*, that which would occur in the absence of any competition, is given by 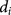. Inter-specific competition among individuals is defined as 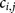 which is the rate at which an individual of species *j* acquires resources, potentially resulting in the death of an individual of species *i*. It is therefore an active process, expressing the ability of an individual to deplete resources and thereby kill (at least indirectly) its neighbours. This rate is uniform in space but with a maximum radius given by 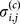. This means that an individual of species *j* selects with equal probability a site within a distance 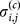 with a rate 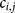. If the chosen site is occupied by an individual of species *i*, that individual dies. The intra-specific competition parameter is a special case equivalent to 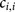. It is the rate at which an individual of species *i* acquires resources, potentially resulting in the death of another individual of species *i*, uniform in space with a maximum radius 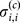. This treatment of interference competition as an active process differs from that more commonly used whereby a focal individual's likelihood of mortality is a function of the number of neighbours, in other words a passive process. The two treatments are mathematically equivalent and can be related by a similarity transformation. For a detailed discussion of the choice of active competition in this study and its implications see S1 Note.

An individual was chosen at random and the probability of a birth or death event calculated according to the corresponding rates. A computing time step was counted each time that on average all individuals of the system had been updated, and a generation was defined as being when on average all individuals had attempted to reproduce once. The simulation was automatically stopped when the change in total community density (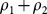) fell below 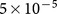 in 20 time steps. This arbitrary criterion was established during preliminary investigations to be a robust indicator of long-term outcomes, ensuring that we were close to the stationary state while reducing the risk of extinctions due to finite size fluctuations [22]. See S1 Fig. for an example of population density changes over time.

We obtained phase diagrams of the coexistence region for systems in which interactions occurred either over long or short distances, or over relatively longer distances for either the common or rare species. This was accomplished by running multiple simulations beginning with values of 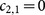, 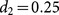 and then with independent increments of 0.01 in each until 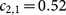, 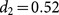. All other parameters remained fixed (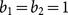, 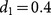, 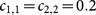, 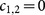). This represents a total of 28,080 simulations for each composite phase diagram. Parameter values were chosen based on prior investigation which had determined that they encompassed the full range of potential outcomes within this system. We found the stationary densities of both species for each set of values and note where they do not fall to zero for either species, i.e. both species are maintained indefinitely. The phase diagrams obtained by keeping {*c*_2,1_, *d*_2_} fixed and altering other parameters have similar properties to those presented here, which are therefore used to illustrate a more general set of principles. Invasion analyses were conducted for a subset of parameter combinations and in all cases confirmed the outcome.

To convey the underlying spatial pattern of individuals we present the pair correlation function 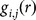, a robust descriptor of spatial pattern structure [23], [24]. It is obtained from the first derivative of Ripley's 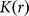 function [25], which gives the expected number of points within a distance 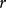 summed across all points in the pattern and divided by its average density 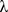. It can be estimated as



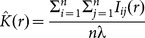
(1)


where *r* is the distance from each point *i*, 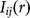 is 1 for each *j* within *r* of *i* and otherwise 0, and *n* is the total number of points. This provides a cumulative function which can be converted to the pair correlation function 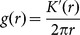. In ecological terms it describes the ‘plant's-eye perspective’ (*sensu* [5]) of neighbourhood density at increasing distance *r*. If densities are independent at a given distance, 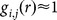. When 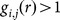, pairs of individuals are more abundant than the spatial average, while 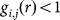 indicates that they are less abundant.

### The Mean-Field Approximation

We begin by defining when two species are expected to coexist if all individuals experience average conditions. These null expectations were obtained from a spatially-explicit extension of the classic Lotka-Volterra competition equations, referred to hereafter as the Mean Field Approximation (MFA), and using the same parameters as in Table 1. The dynamics of the mean density 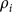 of species *i* can be described as:
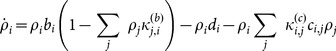
(2)


In this equation 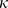 represents the ratio between the observed density of competitors around a given individual and the mean density of the whole system. When 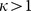, individuals experience a greater density of competitors than would be expected based on average conditions, while the opposite is true for 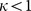. Hence 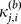 captures the difference from the mean density of competitors of species 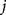 experienced by an individual of species 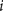 when it attempts to reproduce. Likewise 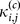 is the difference from the mean density of species 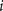 experienced by species 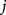 when it obtains resources. This is a useful construct as it quantifies how competition rates change as the result of spatial structure. It is related to the widely-used Ripley's 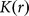 function (equation 1; [25]) since for a given radius 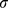 it is equivalent to the value of 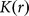 divided by the area of integration. Note that in our study 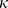 is a state variable and not a parameter, as in some previous treatments. A generalised derivation is provided in S2 Note.

When the spatial structure of the system is not taken into account, the competition experienced by any single individual is the mean competition exerted by all others, irrespective of distance. It is equivalent to the behaviour of a community with homogeneous density equal to the mean of the entire system (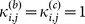). Equation (2) for a community of two species reduces to the well-known Lotka-Volterra model:



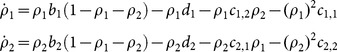
(3)


A stability analysis on the stationary solutions of (3) reveals that the following condition is necessary for coexistence to occur



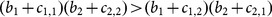
(4)


This is a generalisation of the commonly-stated condition that intra-specific competition must exceed inter-specific [1] but taking into account the reduction of the birth rates due to overall density. When the left hand side of (4) is equal to the right the species are ecologically equivalent and the dynamics will be driven by ecological drift [26].

**Figure 1:**
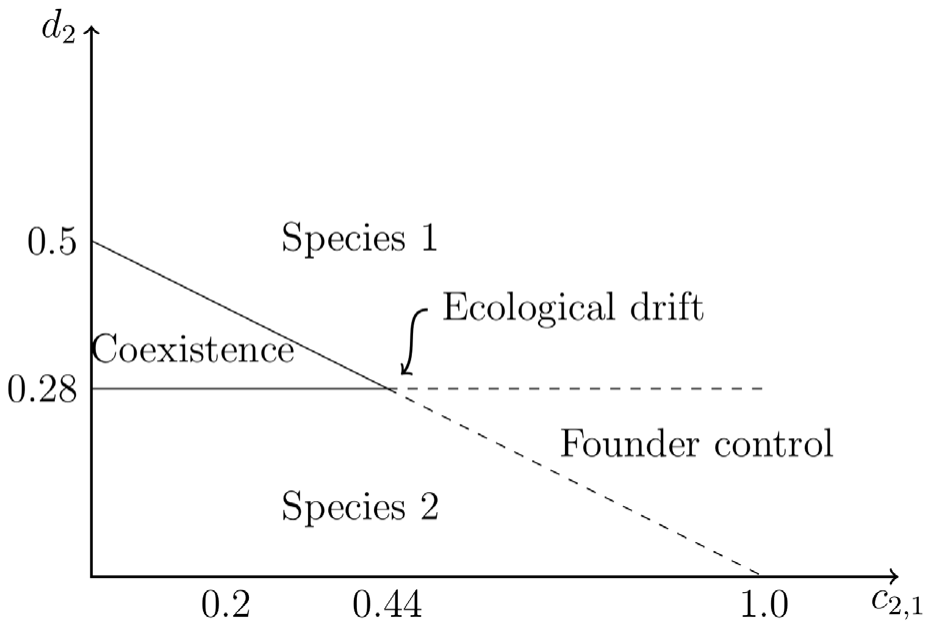
Phase diagram of competition outcomes over values of 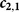 and 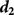 predicted by the Mean Field Approximation (MFA; see Methods for details). All other parameters fixed: 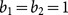, 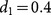, 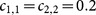, 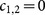.

Based upon these equations, the region within which coexistence occurs for an arbitrary pair of parameters is illustrated in Fig. 1. Similar figures can be generated through the choice of any pair of parameters from Table 1 which include either of the inter-specific competition parameters (*c*_2,1_ or *c*_1,2_) and one with an independent direct effect on the population density of a single species (e.g. its rate of births *b* or death *d*). By varying two parameters, while keeping all others fixed, the model can lead to either competitive exclusion of one species, stable coexistence, or founder control whereby the species with an initial numerical advantage (determined by chance) comes to dominate.

### Ecological pressure and local interactions

It is therefore apparent that whether a species has a greater inter-specific competition rate (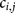) is not a reliable criterion by which to infer its numerical dominance. Instead we propose an additional descriptor of the state of a population which we refer to as the ecological pressure acting upon it. Ecological pressure is defined as the sum of demographic forces that a particular population experiences. The change in density of a population 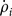 is inversely related to the ecological pressure it experiences 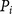, and the units of ecological pressure can be chosen such that 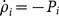. The minus sign comes from the conceptual interpretation of ecological pressure as the forces that the environment exerts over the species (and not the species over its environment). This means that if the ecological pressure over a species in a community is positive the density of that species decreases, and *vice versa* if it is negative.

The spatial Lotka-Volterra model (equation (2)) can be obtained from the following form for the ecological pressure on species *i*:



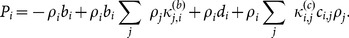
(5)


Ecological pressure may change over time for various reasons. These include fluctuations in the densities of one or more species, variation in external abiotic factors, or even shifts in the spatial organisation of the community. The relationship between 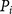 and 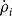 allows inference of the actual ecological pressure in natural systems from measurements of the change in population density over time. This is important because it provides a potential means to assess the performance of a theoretical model by comparing the predicted ecological pressure with the observed demographic change.

Over a finite period of time 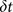, the actual change in population density 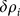 can be found by the series expansion
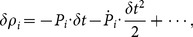
(6)where 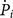 denotes the total time derivative of *P_i_*. The concept of ecological pressure is useful in this study because calculating how it changes as a result of variation in the range or strength of interactions allows us to account for changes in population densities or outcomes of competition. When the system is in a stationary state, i.e. both populations are at equilibrium, the total ecological pressure is zero. By altering the parameters of one or more species we can identify how ecological pressure will change as a result. For example, in a two species system the ecological pressure over species *j* (equation (5)) will be diminished by reducing the inter-specific competition range of the other species (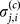), which in turn enables an increase in the population density of species *j*.

## Results

### Model outputs

When compared to the results of spatially-explicit simulations of the individual-based model, the mean-field approximation fails to accurately predict the region of parameter space within which coexistence occurs. This effect is particularly pronounced when interactions occur over short ranges, as expected in nature when individuals are most influenced by their nearest neighbours.

**Figure 2:**
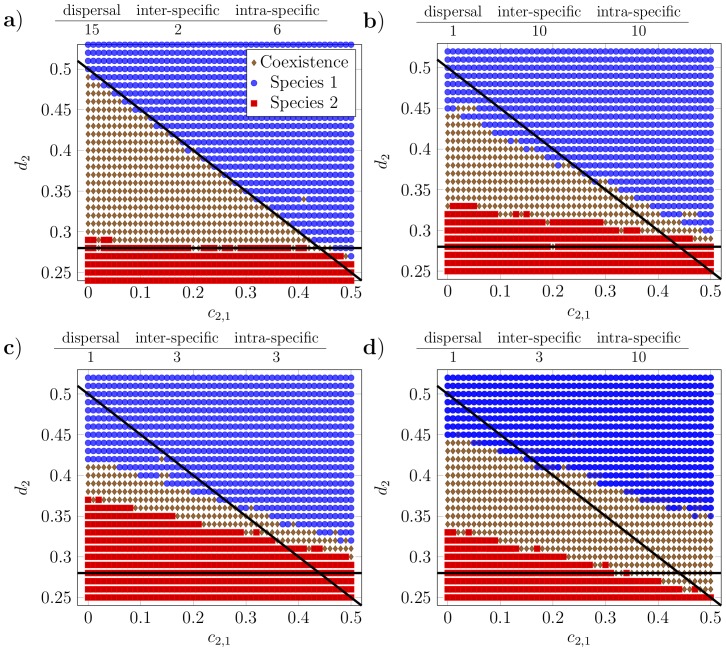
Coexistence diagrams for a system of two species obtained by changing the values of the inter-specific competition rate of species 1 (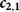) and the intrinsic death rate of species 2 (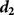). All other parameters fixed at 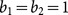, 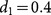, 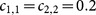, 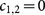. Each point represents the outcome of 20 realisations of the model (see Methods). Blue circles: only species 1 survives; red squares: only species 2 survives; brown rhombus: coexistence. Solid black lines represent predictions based on the mean-field approximation. The table above each diagram shows the values of the range of dispersal 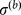, inter-specific and intra-specific competition (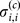, 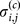). a) long range dispersal; b) localised dispersal; c) short-range interactions with equal values for both species; d) variation in intra- and inter-specific competition ranges among species. Definition of parameters in Table 1; specimen patterns based on c) and d) are shown in S3 Fig.

With long range dispersal, mean-field predictions are almost recovered, regardless of the values of the competition range (Fig. 2a). This is expected since offspring are able to escape from regions of high density, removing the constraints on recruitment imposed by spatial population structure. The parameter space within which stable coexistence occurs becomes smaller when the dispersal range 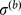 is short, making coexistence less likely (Fig. 2b). This is the result of a reduction in population-level birth rates due to a higher density of individuals near their parents, i.e. the number of successful offspring decreases. Localised dispersal and short range interactions for both species lead to a reduction in the parameter space for coexistence in comparison with long range interactions (Fig. 2c). This is in agreement with a previous mathematical result [27] and occurs because the area from which the parents obtain resources is reduced, making competition in the local neighbourhood more intense, thus reducing even further the number of successful offspring near their parents.

Only when dispersal is highly localised is the region of coexistence modified by the ranges of competitive interactions. Some parameter combinations decrease the potential for coexistence (Fig. 2b, 2c), while others allow coexistence in regions where it was not predicted by the MFA (Fig. 2d). These results are robust to alternative parameter values (e.g. S2 Fig.).

**Figure 3:**
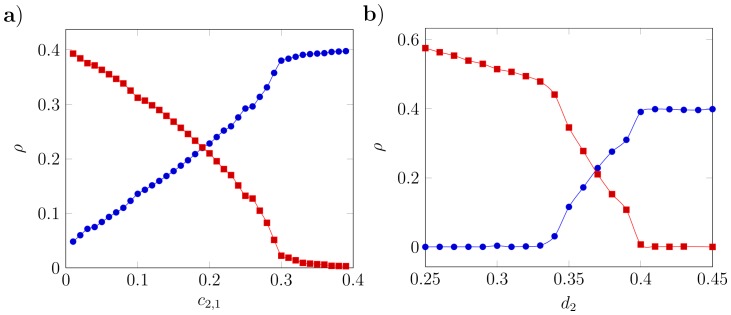
Typical density 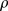 of two species obtained from linear transects through the coexistence diagram in Fig. 2c for the simulated communities. Blue line and circles: species 1; red line and squares: species 2. a) for variation in 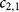 with 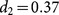, b) for variation in 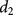 with 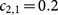. All other parameters as Fig. 1.

The transition from coexistence to competitive exclusion in the simulations occurs due to continuous changes in the densities (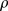) of each species, rather than a sharp boundary (Fig. 3). Fig. 3a demonstrates that there is an important region of parameter space in which despite species 2 being competitively weaker (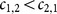) it is numerically dominant (i.e. the red line is above the blue). From Fig. 3b we can see that the susceptibility of a species' population density to changes in its intrinsic death rate is greater (i.e. a steeper slope) when another species is present, even as a minority element. These patterns reinforce our view that complementarity is central to understanding coexistence, since the density of each species responds to the presence of the other; it is not merely the case that an inferior competitor fits around the pattern generated by the stronger species.

**Figure 4:**
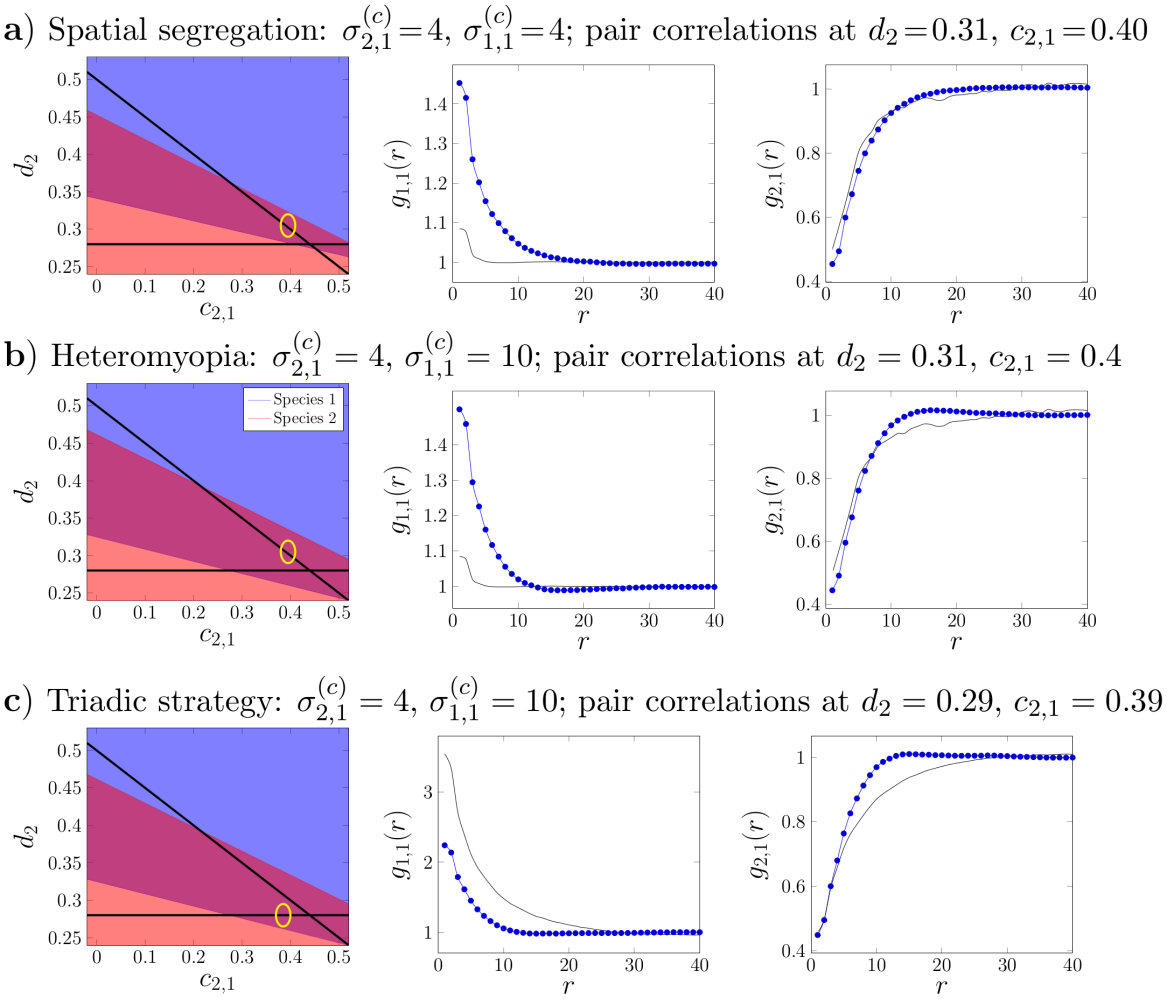
Three examples of spatial coexistence beyond the predictions of the Mean Field Approximation (MFA) achieved through localised interactions. Left column: phase diagrams obtained as in Fig. 2 with predictions of the MFA (solid black lines), though for clarity we omit individual points representing each set of simulations. Circles indicate the point at which corresponding spatial pattern statistics were calculated. Middle column: the intra-specific pair correlation function of species 1, 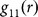, indicating the deviation from average density of individuals of the same species at distance *r* from any single individual. Thin solid line is the identical function but with long-range interactions (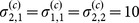), representing the MFA (as in Fig. 2a). Right column: cross-pair correlation function of species 1 to species 2, 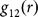. The deviation from the average density of species 2 at a distance *r* as experienced by an individual of species 1 is proportional to the value of this function at *r*. Thin solid line is the cross-pair correlation in the case of long range interactions.

In Fig. 4 we illustrate three cases where spatial coexistence can be achieved through localised interactions in regions where the MFA based upon the expectations of the Lotka-Volterra equations fails to predict it. Each phase diagram represents a particular combination of parameters, while the summary statistics illustrate the intra- and inter-specific pair correlation functions calculated at the point marked on the corresponding phase diagram. By comparing the empirical correlation function with that expected under the MFA we can observe the change in the dimensions of clusters caused by switching from long-range interactions to localised. The correlation functions reveal that each case exhibits a unique spatial pattern, thereby indicating different underlying coexistence mechanisms.

Fig. 4a shows that short-range interactions (localised intra- and inter-specific competition) promote coexistence outside the MFA predictions. Coexistence occurs as a consequence of increased ecological pressure on the numerically-dominant species due to intense intra-specific competition, following from reduction of the range over which this occurs 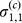. In conjunction there is a reduction in ecological pressure on the rarer species due to shortening of the inter-specific competition range of species 1 (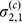). This can be verified by describing the change in ecological pressure for each species from equation 5:
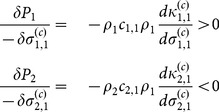
(7)


The phenomenon holds at least up to the first order in a time expansion of the ecological pressure (equation (6)), given that individuals within species are aggregated (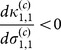) and segregated between (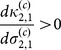). This mechanism lies behind the spatial segregation hypothesis [12], but note that enhanced coexistence is only exhibited near the point of ecologically-equivalent species.

In Fig. 4b the numerically-dominant species 1 has shorter-range inter-specific than intra-specific competition (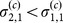), generating an increase in the coexistence region consistent with heteromyopia [18]. The reduction in ecological pressure on the rarer species 2 arises because of the relatively shorter range of inter-specific competition from the numerically-dominant species:
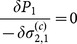


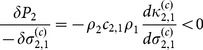
(8)


The effect holds at least up to the first order in time (equation (6)) with segregation between species (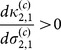). The same process is also present in spatial segregation but the lack of intensified intra-specific competition identifies it as a distinct phenomenon.

Finally, Fig. 4c shows coexistence via an additional mechanism which has not previously been identified. The effect of changing 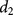 from Fig. 4b to Fig. 4c is to transform species 1 from being numerically dominant to rare. At the lower bound of the coexistence region, short-range inter-specific competition by the rare species reduces the ecological pressure acting upon it. This can be seen from the decrease in height of 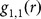 in Fig. 4c (middle column) as a result of shortening 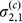. From the perspective of individuals of the rarer species there is a reduction of conspecifics in its neighbourhood (i.e. reduced clustering), thereby reducing intra-specific competition. This behaviour can only be predicted via the second order term in equation (6), meaning it is observed only once a second generation is born (grandchildren). For this reason we refer to it as a triadic mechanism; its elucidation depends on third order spatial moments, i.e. a minimum of three individuals.

This can be seen by demonstrating that there is no immediate change in the ecological pressure on species 1 as a result of changing 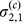 (see equation (8)). This means that the simple linear term in the time expansion of ecological pressure cannot account for the coexistence region in Fig. 4c. Only the second order in time of equation (6) can account for this effect:
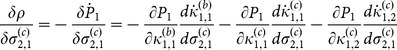
(9)


In general 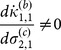 and 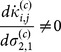. For a further demonstration based on spatial moments see S3 Note. To summarise, in the triadic mechanism it is the ability of grandchildren to escape the competition of their grandparents which enables the persistence of the rarer species. Note that it is the characteristics of the minority species which dictate whether coexistence occurs.

## Discussion

Our simulations determine the conditions under which coexistence between two sessile species within a uniform two-dimensional environment is expected to occur. We have combined two existing mechanisms for spatial coexistence, the spatial segregation and heteromyopia hypotheses [12], [18], into a single scheme, resolving an apparent contradiction between them regarding whether clustering acts to maintain or prevent coexistence and determining the circumstances in which each applies. Furthermore we have revealed an additional mechanism for spatial coexistence which has not been previously recognised, which we describe as a triadic mechanism, and which depends upon the characteristics of the minority species rather than relying upon opportunities provided by the dominant.

A common feature of theory, simulation models and experiments in this field to date has been an assumption that one species is competitively dominant (i.e. causes greater reductions in standing levels of a resource [2]), and the apparent paradox is the continued coexistence of an inferior competitor. In contrast, we contend that coexistence should be seen as a two-sided process; understanding the structure of a mixed community requires an integration of the complementary forces structuring the spatial patterns of all participants, achieved by determining the ecological pressure acting upon each of them. This altered perspective allows for a reconciliation of apparently conflicting predictions.

The novel triadic mechanism is an emergent effect of dynamic changes in the spatial structure of the community across multiple generations rather than a direct escape from competition. Reducing the range of inter-specific competition of the rare species (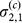) alters the community spatial structure, which through the consequent reorganisation of spatial patterns reduces the ecological pressure on itself. Fig 4c shows that the triadic mechanism promotes a less clustered distribution of the rare species, thereby reducing intra-specific competition (as a result of reducing local densities of conspecifics 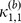 and 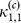). The more homogeneous distribution of the rare species occurs because reducing its inter-specific competition range allows the other species to penetrate its clusters. The competition that the dominant species exerts over the rare species does not increase greatly because it is already long-ranged, thus the relative distance between individuals of the two species has a limited influence on coexistence.

The change in pattern is caused by the locations into which offspring successfully recruit relative to their parents, which differ from those expected in the presence of long range inter-specific competition. A minimum of three generations — and thus three individuals — is required to observe this mechanism (see S4 Note for a mathematical demonstration). The degree of competition between pairs does not alter appreciably, but rather the joint competitive effect of two ancestors on a third-generation offspring is reduced. Hence we refer to it as a triadic mechanism. In addition, the time expansion of the ecological pressure allows each moment equation from the hierarchy of dynamics [28], [29] to be related with a time-related hierarchy of stages in the development of a population, where the immediate changes can be described with only the dynamic equations of the first moment, but a description of later stages requires the dynamic equations of the second moments and so forth (see S3 Note for a demonstration utilising second order moments).

The signal of the triadic mechanism can be detected via characteristic changes in the shapes of the pair correlation functions (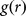 in Fig. 4c) which distinguish it from the two alternatives. It is predicted to apply when the rarer species is the stronger resource competitor and each individual competes with members of its own species over greater distances. Note that it cannot be assumed that the dominant competitor for resources is necessarily more numerous; in many cases a dominant resource competitor can be outnumbered or even excluded by a subordinate species (see Fig. 3b and [30]).

The triadic mechanism is the first to imply the importance of third-order spatial correlations in coexistence, or three generations from a dynamic point of view. The reduced range of inter-specific competition in the rare species does not directly reduce the ecological pressure acting upon it, but only once its clusters reduce in size as a result. The mechanism differs from the familiar competition-colonisation trade-off [9] as the ability of the numerically subordinate species to persist occurs as a result of the rearrangement of the community spatial structure and not due to a strict phenotypic trade-off.

This demonstrates that simplistic views of individual interactions based on a snapshot within in time, or from assessing the survival of offspring, are not always sufficient to account for the behaviour of ecological communities. Previous approaches to modelling spatially-structured systems have employed moment equations which assume that third-order effects are trivial (e.g. [30], [31]). Our work demonstrates that these cannot be dismissed, and sometimes it is necessary to take into account interactions among more than two individuals. In this sense coexistence can be regarded as an emergent property of spatially-structured systems, the phenomenon depending upon complementary combinations of traits in each species.

Results consistent with the triadic mechanism were obtained by [32] from field data on seven species of annual plants in grasslands over two seasons. The larger-seeded species, capable of exploiting resources over a greater area, were the most strongly recruitment-limited, and thus tended to be numerically subordinate. The authors concluded that the competition-colonisation trade-off, which would also be consistent with these characteristics, was not sufficient to maintain coexistence in this system. Notably two of the species reduced their degree of clustering over two years, matching the unique predictions of the triadic mechanism.

A related mechanism for multi-species coexistence is heteromyopia, where simulation modelling predicts that increased spatial segregation of competing dominants creates interstices in their pattern which can be exploited by an invading species [18]. This also depends upon the range over which competition occurs being greater within than between species. In this case, however, it is the numerically-dominant species which exhibits this property. Few have looked for evidence of the underlying assumption; in the only study of which we are aware, no evidence was found in support [33]. The resulting patterns differ from those predicted via the triadic mechanism (see Fig. 4b and S4 Fig.). Heteromyopia is important only when the dominant species has a shorter inter-specific competition range than intra-specific. In contrast, the triadic mechanism operates when the inter-specific competition range of the subordinate species is shorter than that of the dominant species. Heteromyopia acts by reducing the radius of the clusters formed by the more abundant species, while its competitor remains tightly aggregated. This allows it to be distinguished from the triadic mechanism which leads to a more even distribution of the rarer species, though in principle both could act simultaneously.

Finally, the spatial segregation hypothesis [12] is confirmed as enabling coexistence, though only for species close to the point of equivalence in their life history parameters. Previous mathematical results [27] are compatible with a slightly modified version of the original hypothesis, showing that finite dispersal and localised interactions lead to spatial structure that enhances the diversity of similar species. Even when species are effectively identical, once some process such as limited dispersal has created aggregations, these can stabilise despite an absence of environmental variation, allowing competitive exclusion to be almost indefinitely deferred [34]. Aggregation is further reinforced by mortality of isolated individuals [34], [35]. Short-range dispersal is itself insufficient to generate coexistence, and in fact tends to reduce its likelihood (Fig. 2c). The more similar any two species are, the more comparable are the levels of intra-specific competition which they experience through clustering. Hence neither gains a strong competitive advantage and the reduction in ecological pressure through segregation outweighs any increase from clustering.

Assessment of the ecological pressure acting on each species reveals that spatial segregation and heteromyopia both cause similar reductions in competition among species but spatial segregation is the only one to also increase within-species competition. It is therefore the only case in which self-limitation through spatial structuring can truly be said to enhance coexistence.

The effectiveness of spatial segregation in preventing competitive exclusion has been demonstrated for sessile organisms in multiple studies, improving survival of inferior competitors [15], [16] and usually favouring the minority species [14], [17], though not always [13]. Note however that clustering is not always beneficial; as [30] demonstrates, a species can drive itself locally extinct through strong competition within clusters, even when it is able to persist in monoculture. In experimental trials with plants the strongest competitor suffered the greatest penalty when clustered [15].

Within our simulations, coexistence is defined as joint stochastic boundedness, such that both populations are expected to persist indefinitely [36]. Coexistence is evident from the systems having reached a stable state. In the field, however, confirming coexistence requires evidence of some process enabling a species to persist despite the presence of its competitor, distinguishing it from mere co-occurrence of non-interacting species [37]. The usual test is the ability of each species to increase when rare, often known as the invasibility criterion [1], [37]. Nevertheless, while invasibility is indicative, it is not on its own a satisfactory criterion for determining stochastic boundedness of interacting populations (and indeed can be violated [36] or even reversed [30]). This is problematic as invasibility has become synonymous with coexistence in many treatments, despite the likelihood of Allee effects overriding invasion by small initial populations. A further finding from Fig. 3 is that transitions from coexistence to monodominance occur through smooth changes in the density of each species and do not form sharp boundaries. This demonstrates that there is a large region of parameter space within which a rare species can stably coexist without increasing its density. The default state of a community is not the equal abundance of all species.

Individual-based models have great potential to provide new insights into ecological theory and to advance long-established fields of study [38]. Most theoretical studies to date (e.g. [18], [39]) have examined the passive reponses of individuals to neighbourhood density, in contrast to our models, which employ an active view of competition, documenting the resource acquisition of organisms. In S1 Note we show the relation of the parameterisation employed in previous works with that used here.

Competition in our system occurs for both space and an unspecified and unmodelled resource which is obtained locally by individuals. While this can be visualised as a plant obtaining nutrients or water from soil, indirect processes such as apparent competition via shared natural enemies are able to generate long-range interactions beyond the reach of any individual [10]. We do not consider the case where interactions might be facilitative, which would further increase the scope for multi-species coexistence. Likewise, while adaptive speciation provides additional opportunities for coexistence [40], we assume that this takes place on a longer timescale than considered here. It is noteworthy in this context that some plant species seem to be least affected by competition with those species which are more frequent neighbours — including conspecifics — suggesting adaptation to spatial patterning [41]. Further potential for spatial coexistence is provided when species have specialised resources that are distributed unevenly through either space or time [10], [11]. Finally, in real systems, the role played by spatial patterning can itself change through time in response to shifting environmental conditions [14]. These findings are not inconsistent with our model, which can be seen as presenting the minimum conditions for spatial coexistence.

Our simulation study of spatial coexistence has combined existing theory into a common framework and described for the first time a previously unrecognised triadic mechanism whereby multiple species can co-occur in perpetuity. This is the emergent outcome of interactions among individuals across three generations. Through ecological pressure we have demonstrated that spatial coexistence arises due complementary combinations of species traits rather than purely through self-limitation of dominant competitors. In particular, the triadic mechanism depends upon the traits of the rare species. Further tests are required to examine whether these effects occur in nature; our models provide clear expectations for the observed spatial patterns and resultant population dynamics. We anticipate that the parameters of our system will be both intuitive and tractable, and look forward to experimental tests of the principle of spatial complementarity.

## Supporting Information

S1 Fig **Typical trajectory of population densities in a simulated two species community prior to reaching a stationary state.**
(TIFF)

S2 Fig
**Stability of the results to changes in competition intensity.** Coexistence diagrams with 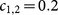; all other parameters identical to Fig. 2 in Results. Dotted line at 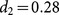 indicates the mean field coexistence boundary for 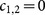 from Fig. 2. All coexistence mechanisms apply in the appropriate regions of parameter space, with an identical increase in (d) due to effects of spatial structure. This holds regardless of the value of 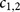 chosen.(TIFF)

S3 Fig
**Typical specimen patterns based upon iterations of the parameter combinations shown in a)** **Fig. 2c and b** **)** **Fig. 2d** **with** 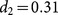 **,** 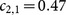 **.** Blue: species 1; red: species 2.(TIFF)

S4 Fig **Pair correlation functions for the a) numerically-dominant species and b) rarer species when either heteromyopia or the triadic mechanism is present.** Heteromyopia reduces the radius of the clusters formed by the dominant species (left), reducing inter-specific competition. The triadic mechanism promotes a more uniform distribution of the subordinate species (right) reducing intra-specific competition.
(TIFF)

S1 Note **Active versus passive representation of the Lotka-Volterra equations.**
(PDF)

S2 Note **General derivation of the spatial Lotka-Volterra model.**
(PDF)

S3 Note **Ecological pressure and spatial moments.**
(PDF)

S4 Note **Ecological pressure, competition across generations and the triadic mechanism.**
(PDF)
