# Supplementary figures and images for: Spatial Complementarity and the Coexistence of Species
Source: PLoS One. 2014 Dec 22;9(12):e114979. doi: 10.1371/journal.pone.0114979 (PMC4274010; doi:10.1371/journal.pone.0114979)

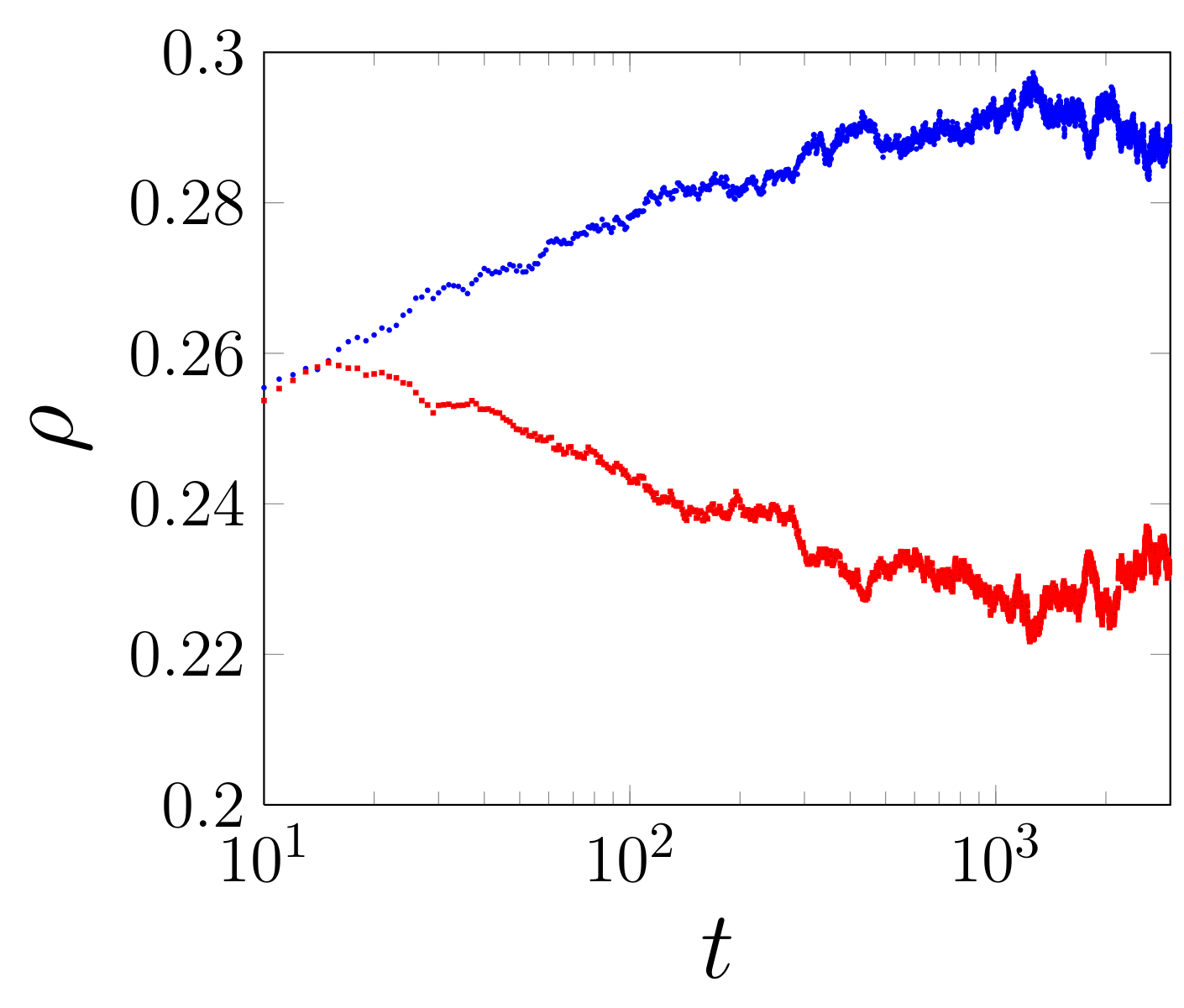

Supplement: S1 Fig — Typical trajectory of population densities in a simulated two species community prior to reaching a stationary state. (TIFF) [file pone.0114979.s001.tiff]

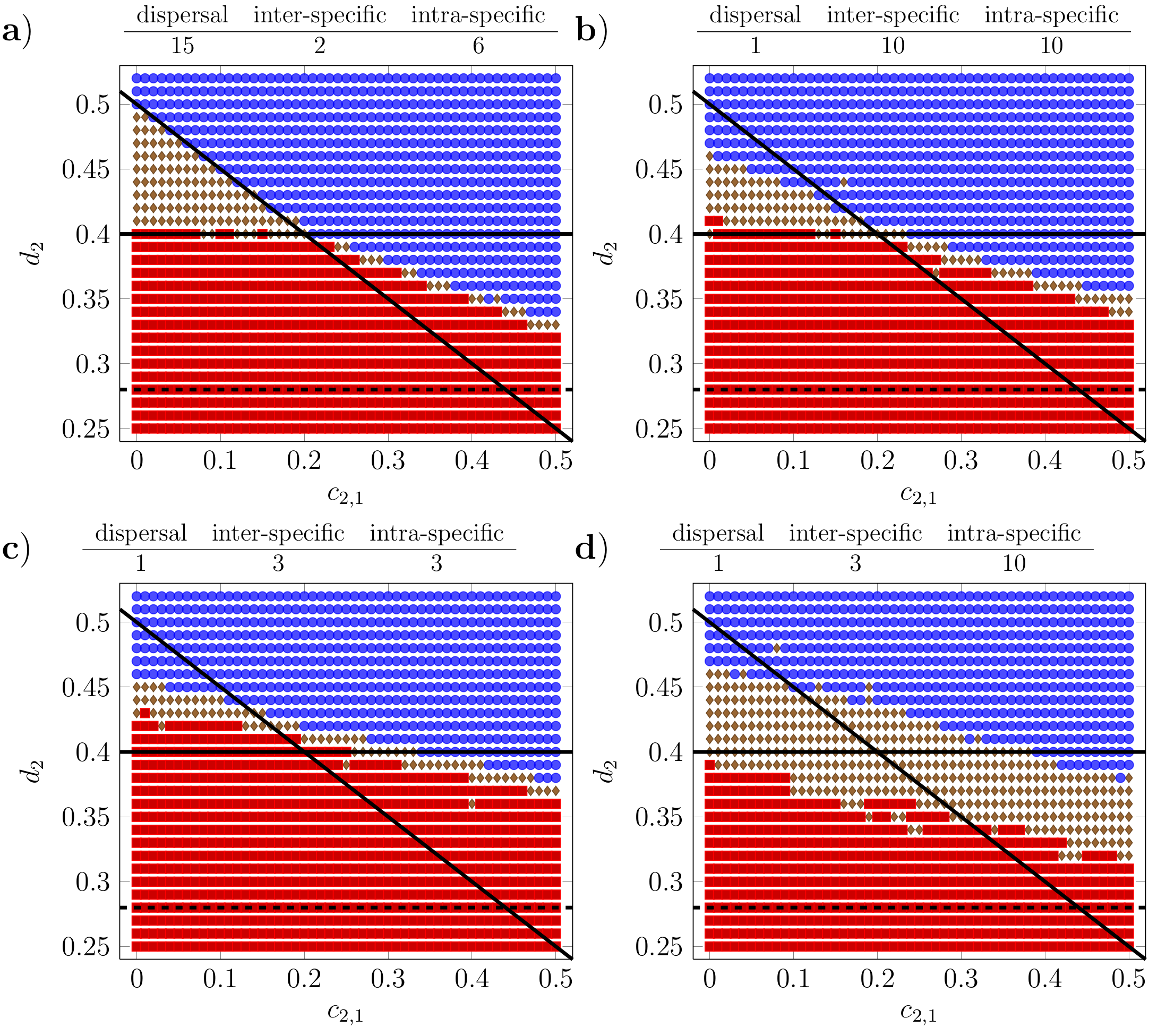

Supplement: S2 Fig — Stability of the results to changes in competition intensity. Coexistence diagrams with ; all other parameters identical to Fig. 2 in Results. Dotted line at indicates the mean field coexistence boundary for from Fig. 2. All coexistence mechanisms apply in the appropriate regions of parameter space, with an identical increase in (d) due to effects of spatial structure. This holds regardless of the value of chosen. (TIFF) [file pone.0114979.s002.tiff]

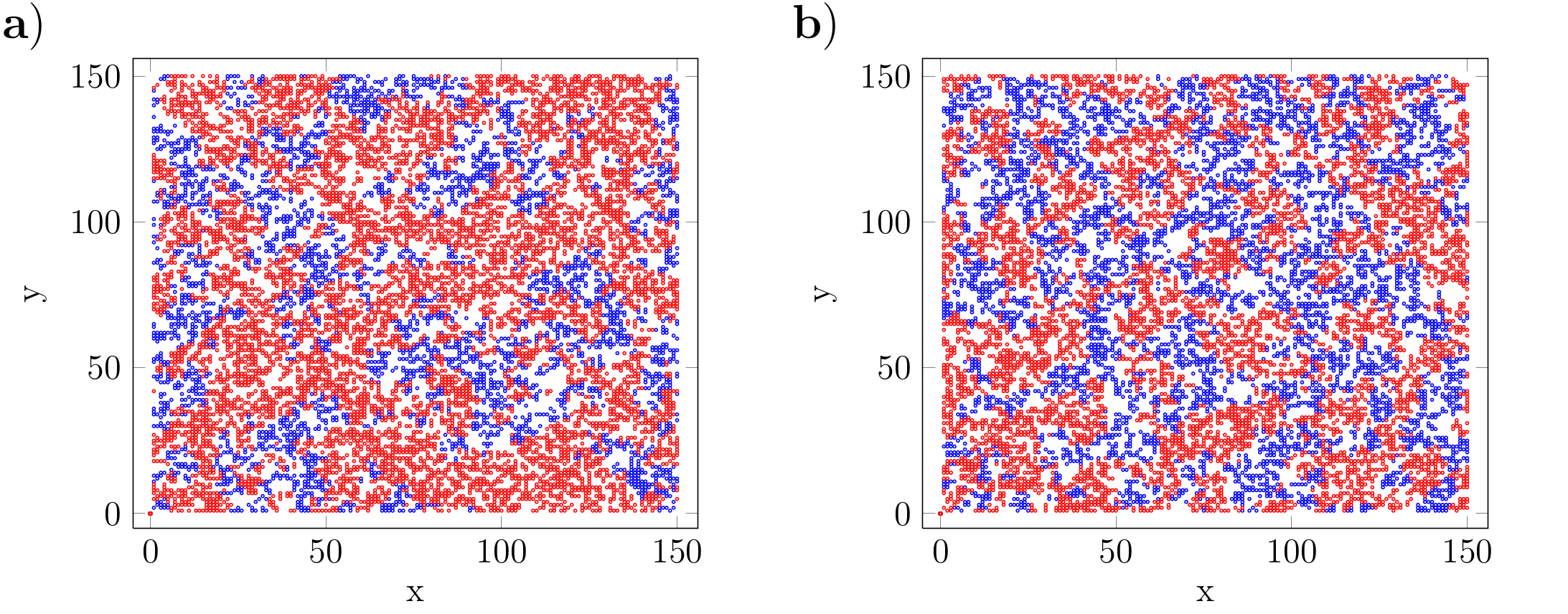

Supplement: S3 Fig — Typical specimen patterns based upon iterations of the parameter combinations shown in a) Fig. 2c and b ) Fig. 2d with , . Blue: species 1; red: species 2. (TIFF) [file pone.0114979.s003.tiff]

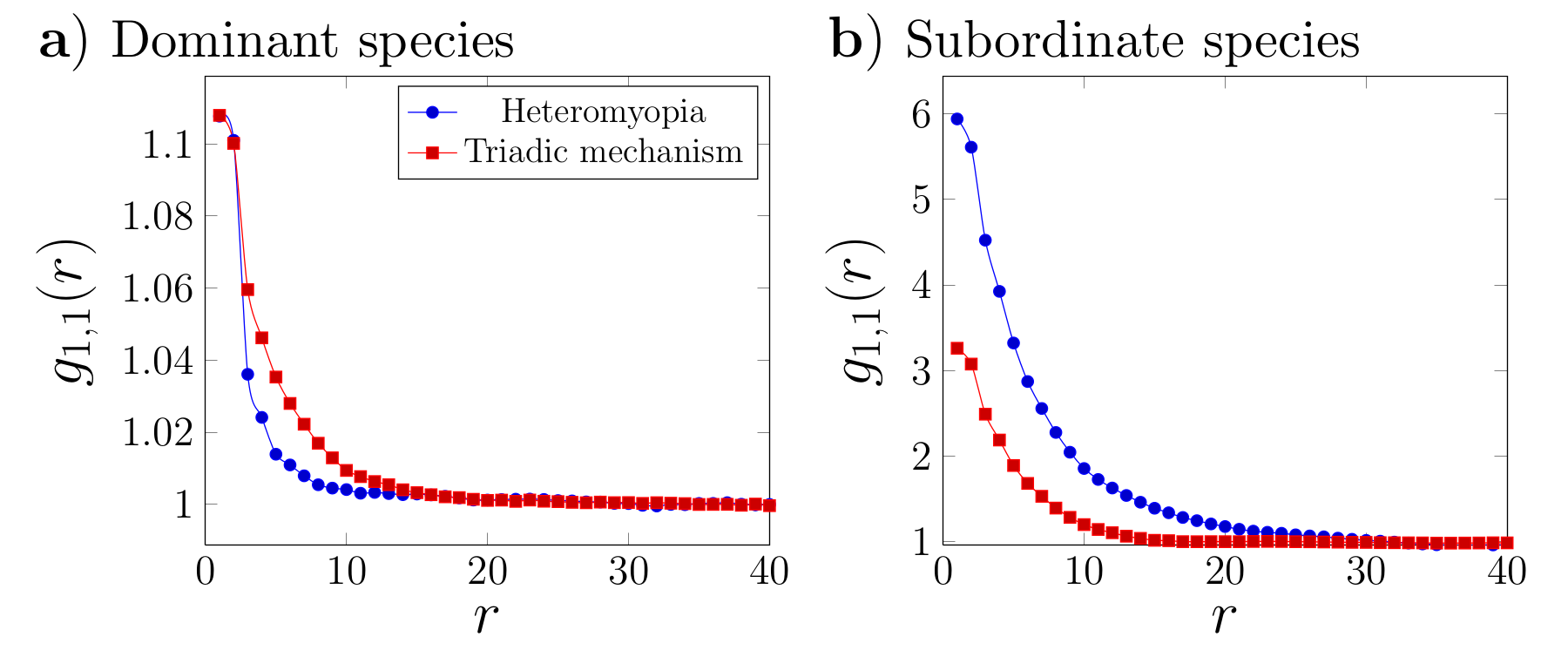

Supplement: S4 Fig — Pair correlation functions for the a) numerically-dominant species and b) rarer species when either heteromyopia or the triadic mechanism is present. Heteromyopia reduces the radius of the clusters formed by the dominant species (left), reducing inter-specific competition. The triadic mechanism promotes a more uniform distribution of the subordinate species (right) reducing intra-specific competition. (TIFF) [file pone.0114979.s004.tiff]
