# Supplementary material for: Spatial Complementarity and the Coexistence of Species
Source: PLoS One. 2014 Dec 22;9(12):e114979. doi: 10.1371/journal.pone.0114979 (PMC4274010; doi:10.1371/journal.pone.0114979)
Supplement: S1 Note — Active versus passive representation of the Lotka-Volterra equations. (PDF) [file pone.0114979.s005.pdf]

Supplementary Note S1

Active versus passive representation of the Lotka-Volterra equations

In previous studies [3–6] competition was defined as $d_{i,j}$, the rate at which an individual of a species i dies because of the presence of an individual of species j. This rate is not the same as our $c_{i,j}$, which represents the rate at which an individual of species j looks for resources common to species i. If $w_{i,j}(\zeta)$ is the competition kernel of species j over species i, and $C_{i,j}(\zeta)$ is the density of species j as it appears to species i, then in the original heteromyopia model [6] the competition terms are expressed as $d_{i,j}I_{i,j}$ with $I_{i,j} = \int w_{i,j}(\zeta)C_{i,j}(\zeta)d\zeta$. In our parameterisation, making use of a similar notation as in previous papers [4, 6], the competition terms are expressed as $c_{i,j}I'_{i,j}$ with $I'_{i,j} = \int w_{i,j}(\zeta)C_{j,i}(\zeta)d\zeta$.

An approximate value of $d_{i,j}$ in terms of $c_{i,j}$ is given by $d_{i,j} = c_{i,j}/A^{(c)}_{i,j}$, where $A^{(c)}_{i,j}$ is the area obtained from the mean distance over which species j interacts with species i. This is so because $c_{i,j}$ is the rate at which species j looks for resources, and the resources that it takes are distributed within the area in which its interactions take place, i.e. the rate $c^{(c)}_{i,j}$ is related to the resources *per capita* that the species requires.

The parameterisation of d could be thought of as a passive representation, while that used in our study is an active representation. The rate $d_{i,j}$ is passive because it is related to how the individual of species i experiences the competition exerted by other individuals. The interaction ranges are defined by the range over which the individual of species i is susceptible to the presence of individuals of species j.

In contrast, the rate $c_{i,j}$ is an active representation, related to the competitive force that individuals of species j exert over species i. The interaction ranges in this representation are related to the distance over which individuals of species j acquire resources. An important difference in model behaviour is that with the active representation, increasing the radius of competition doesn't increase the net competitive effect exerted by an individual, whereas in the passive representation of [6] a greater radius implies on average an influence on a larger number of neighbours. This means that the total impact of an individual increases with the radius over which it acquires resources, causing the two parameters involved in competitive interactions (distance and resource requirements) to no longer be independently controlled. In some instances it is possible to relate different stochastic processes by a similarity transformation [7–9], and the active and passive representations are one such case. This means that they are fully equivalent if their rates are chosen appropriately, i.e. $d_{i,j} = c_{i,j}/A^{(c)}_{i,j}$.

We have selected the active representation because its known algorithmic implementation is more

computationally efficient than the passive representation, in addition to the conceptual advantages. A major conceptual difference from the original heteromyopia model [6] is that increasing the radius of competitive interactions effectively reduces their intensity, since the rate at which an individual acquires resources remains the same though distributed over a greater area. This makes it easier to separate the effects of an increase in competition radius from the net increase in competition across the system as a whole.

As a result of the active representation of competition, our simulations do not employ the Gillespie algorithm for updating individuals, as has been more common in previous work on IBMs in plants. Our choice to make selection of individuals entirely random is a straightforward approach which is commonly recommended for spatially-explicit reaction-diffusion problems (e.g. [10]). The Gillespie algorithm is more suitable for simulations which use a passive representation of competition since it increases the likelihood of selecting an individual with many neighbours. In our simulation, individuals within crowded neighbourhoods will automatically die more often because there are more neighbours capable of killing them. Therefore even if each individual is chosen with equal probability, individuals in clusters have a higher probability of affecting another individual. The particular algorithm chosen should not affect the results; see [11] for a description of many different methods employed to simulate spatially-explicit reaction-diffusion problems and their recommended applications.

References

1. Ripley B (1977) Modelling spatial patterns. Journal of the Royal Statistical Society Series B 39: 172–212.

2. Haase P (1995) Spatial pattern analysis in ecology based on Ripley's K-function: Introduction and methods of edge correction. Journal of Vegetation Science 6: 575–582.

3. Iwasa Y, Nakamaru M, Levin S (1998) Allelopathy of bacteria in a lattice population: competition between colicin-sensitive and colicin-producing strains, Evolutionary Ecology 12: 785-802.

4. Dieckmann U, Law R (2000) Relaxation Projections and the Method of Moments. In: Dieckmann U, Law R, Metz J, editors, The Geometry of Ecological Interactions: Simplifying Spatial Complexity, Cambridge University Press. pp. 412–455.

5. Neuhauser C, Pacala S (1999) An explicitly spatial version of the Lotka-Volterra model with interspecific competition. Annals of Applied Probability 9: 1226–1259.

6. Murrell D, Law R (2003) Heteromyopia and the spatial coexistence of similar competitors. Ecology Letters 6: 48–59.

7. Simon H (1995) Concentration for one and two species one-dimensional reaction-diffusion systems. Journal of Physics A 28: 6585-6603.

8. Santos J (1997) The duality relation between Glauber dynamics and the diffusion-annihilation model as a similarity transformation. Journal of Physics A 30: 3249-3259.

9. Henkel M, Orlandini E, Santos J (1997) Reaction-diffusion processes from equivalent integrable quantum chains. Annals of Physics 259: 163-231

10. Hinrichsen H (2000) Non-equilibrium critical phenomena and phase transitions into absorbing states. Advances in Physics 49: 815–958.

11. Burrage K, Burrage PM, Leier A, Marquez-Lago T, Nicolau DVJ (2011) Stochastic Simulation for Spatial Modelling of Dynamic Processes in a Living Cell. In: Koeppl H, Densmore D, Setti G, di Bernardo M, editors, Design and Analysis of Biomolecular Circuits, Springer. pp. 43–62.
