# Supplementary material for: Spatial Complementarity and the Coexistence of Species
Source: PLoS One. 2014 Dec 22;9(12):e114979. doi: 10.1371/journal.pone.0114979 (PMC4274010; doi:10.1371/journal.pone.0114979)
Supplement: S2 Note — General derivation of the spatial Lotka-Volterra model. (PDF) [file pone.0114979.s006.pdf]

Supplementary Note S2

General derivation of the spatial Lotka-Volterra model

The mean population dynamics of species i with abundance N under mean-field conditions are described by the following general equation:

$$\dot{N}_i = N_i b_i \left(1 - \sum_j \rho_{j,i}^{eff(b)}\right) - N_i d_i - \sum_j \rho_{i,j}^{eff(c)} c_{i,j} N_j \tag{1}$$

where $\rho_{j,i}^{eff(b)}$ is the mean density of species j that on average an individual of species i experiences when attempting to disperse, and $\rho_{i,j}^{eff(c)}$ is the mean density of species i that on average an individual of species j experiences as a result of competitive interactions.

For a generalised dispersal kernel $w_{j,i}^{(b)}(r)$, $\rho_{j,i}^{eff(b)}$ can be expressed as

$$\rho_{j,i}^{eff(b)} = \rho_j \kappa_{j,i}^{(b)} \tag{2}$$

where $\kappa_{j,i}^{(b)} = 2\pi \int w_{j,i}^{(b)}(r) g_{j,i}(r) r dr$ and $g_{j,i}(r)$ is the pair correlation function (see equation (1) in Methods). Likewise $\rho_{i,j}^{eff(c)}$ can be expressed as

$$\rho_{i,j}^{eff(c)} = \rho_i \kappa_{i,j}^{(c)} \tag{3}$$

where $\kappa_{i,j}^{(c)} = 2\pi \int w_{i,j}(r) g_{i,j}(r) r dr$ and $w_{i,j}(r)$ is the competition kernel.

Dividing by the field area and substituting (2) and (3) in (1), we find an explicit spatial version of the Lotka-Volterra model (as equation (2) in Methods):

$$\dot{\rho}_i = \rho_i b_i \left(1 - \sum_j \rho_j \kappa_{j,i}^{(b)}\right) - \rho_i d_i - \rho_i \sum_j \kappa_{i,j}^{(c)} c_{i,j} \rho_j \tag{4}$$

The κ values hold information regarding how the effective density deviates from the mean density. κ can also be seen as the factor that quantifies how the competition rates change due to the spatial structure.

In our simulations $w_{j,i}^{(b)}(r) = \Theta(\sigma_{j,i}^{(b)} - r)/(\sigma_{j,i}^{(b)})^2\pi$ and $w_{i,j}^{(c)}(r) = \Theta(\sigma_{i,j}^{(c)} - r)/(\sigma_{j,i}^{(c)})^2\pi$, where $\Theta(r)$ is defined to be 1 if $r \geq 0$ and 0 otherwise. This makes κ equivalent to Ripleys $K(r)$ function [1,2] divided by the area of integration, i.e. $\kappa_{j,i}^{(b)} = K(\sigma_{j,i}^{(b)})/(\sigma_{j,i}^{(b)})^2\pi$ and $\kappa_{i,j}^{(c)} = K(\sigma_{i,j}^{(c)})/(\sigma_{i,j}^{(c)})^2\pi$. The more generalised

derivation provided above allows for alternative kernels for either dispersal or competition to be fitted.

References

1. Ripley B (1977) Modelling spatial patterns. Journal of the Royal Statistical Society Series B 39: 172–212.

2. Haase P (1995) Spatial pattern analysis in ecology based on Ripley's K-function: Introduction and methods of edge correction. Journal of Vegetation Science 6: 575–582.
