# Supplementary material for: Spatial Complementarity and the Coexistence of Species
Source: PLoS One. 2014 Dec 22;9(12):e114979. doi: 10.1371/journal.pone.0114979 (PMC4274010; doi:10.1371/journal.pone.0114979)
Supplement: S3 Note — Ecological pressure and spatial moments. (PDF) [file pone.0114979.s007.pdf]

Supplementary Note S3

Ecological pressure and spatial moments

First we note that for the competition kernel used in our simulations

$$\dot{\kappa}_{i,j}^{(c)} = \frac{d}{dt}\frac{2}{(\sigma_{i,j}^{(c)})^2}\int_0^{\sigma_{i,j}^{(c)}} g_{i,j}(r)rdr = \frac{2}{(\sigma_{i,j}^{(c)})^2}\int_0^{\sigma_{i,j}^{(c)}} \dot{g}_{i,j}(r)rdr \tag{1}$$

from which we deduce that $\dot{\kappa}_{i,j}^{(c)}$ and in a similar way $\dot{\kappa}_{1,1}^{(b)}$ are functions of the rate of change of the second spatial moment $\dot{g}_{i,j}(r)$. It has previously been shown that the rate of change of the second spatial moment depends on the first, second, and third moments [1], i.e.

$$\dot{g}_{i,j} = F_{\{\sigma_i^{(b)}\},\{\sigma_{i,j}^{(c)}\}}[\{\rho_i\},\{g_{i,j}\},\{g_{i,j,k}\}] \tag{2}$$

where F is a function of the species densities $\{\rho_i\} := (\rho_1, \rho_2)$, the pair correlations $\{g_{i,j}\} := (g_{1,2}, g_{2,1}, g_{1,1}, g_{2,2})$ and the third order spatial correlations $\{g_{i,j,k}\} := (g_{1,1,1}, g_{1,1,2}, g_{1,2,1}, ...)$. F has as parameters the ranges of dispersal $\{\sigma_i^{(b)}\}$ and competition $\{\sigma_{i,j}^{(c)}\}$.

In turn $\dot{\kappa}_{i,j}^{(c)}$ and $\dot{\kappa}_{1,1}^{(c)}$ depend on the first, second, and third moments,

$$\begin{aligned} \dot{\kappa}_{i,j}^{(c)}[\dot{g}_{i,j}] &= F^c_{\{\sigma_i^{(b)}\},\{\sigma_{i,j}^{(c)}\}}[\{\rho_i\},\{g_{i,j}\},\{g_{i,j,k}\}] \\ \dot{\kappa}_{1,1}^{(b)}[\dot{g}_{1,1}] &= F^b_{\{\sigma_i^{(b)}\},\{\sigma_{i,j}^{(c)}\}}[\{\rho_i\},\{g_{i,j}\},\{g_{i,j,k}\}] \end{aligned} \tag{3}$$

and hence their derivatives with respect to $\sigma_{1,2}^{(c)}$ are also functions of the first, second and third moments,

$$\begin{aligned} \frac{d\dot{\kappa}_{i,j}^{(c)}}{d\sigma_{2,1}^{(c)}} &= \frac{dF^c_{\{\sigma_i^{(b)}\},\{\sigma_{i,j}^{(c)}\}}}{d\sigma_{2,1}^{(c)}}[\{\rho_i\},\{g_{i,j}\},\{g_{i,j,k}\}] \\ \frac{d\dot{\kappa}_{1,1}^{(b)}}{d\sigma_{1,2}^{(c)}} &= \frac{dF^b_{\{\sigma_i^{(b)}\},\{\sigma_{i,j}^{(c)}\}}}{d\sigma_{2,1}^{(c)}}[\{\rho_i\},\{g_{i,j}\},\{g_{i,j,k}\}] \end{aligned} \tag{4}$$

In the specific context of the triadic mechanism for spatial coexistence, we therefore conclude that the change in ecological pressure on species 1 due to a change in $\sigma_{1,2}^{(c)}$ can only be accounted for through the second order in the time expansion (6) and that the magnitude of this effect depends on the first, second,

and third moments.

References

1. Dieckmann U, Law R (2000) Relaxation Projections and the Method of Moments. In: Dieckmann U, Law R, Metz J, editors, The Geometry of Ecological Interactions: Simplifying Spatial Complexity, Cambridge University Press. pp. 412–455.
