# Supplementary material for: Spatial Complementarity and the Coexistence of Species
Source: PLoS One. 2014 Dec 22;9(12):e114979. doi: 10.1371/journal.pone.0114979 (PMC4274010; doi:10.1371/journal.pone.0114979)
Supplement: S4 Note — Ecological pressure, competition across generations and the triadic mechanism. (PDF) [file pone.0114979.s008.pdf]

Supplementary Note S4

Ecological pressure, competition across generations and the triadic mechanism

Here we identify the role of inter-generational competition in maintaining coexistence through the triadic mechanism. In order to simplify the notation, all the sub-indexes have been omitted to express that the corresponding symbol is in a vector form, i.e.

$$
\begin{aligned}
P &:= (P_1, P_2)\\
\rho &:= (\rho_1, \rho_2)\\
\kappa &:= (\{\kappa_{i,j}^{(b)}\}, \{\kappa_{i,j}^{(c)}\})\\
\frac{\partial}{\partial \rho} &:= \left(\frac{\partial}{\partial \rho_1}, \frac{\partial}{\partial \rho_2}\right)\\
\frac{\partial}{\partial \kappa} &:= \left(\left\{\frac{\partial}{\partial \kappa_{i,j}^{(b)}}\right\}, \left\{\frac{\partial}{\partial \kappa_{i,j}^{(c)}}\right\}\right)
\end{aligned}
$$

and $A \cdot B$ denotes the product of A and B. If $A_{i,j}$ is an arbitrary quantity with sub-indices i, j, then $\{A_{i,j}\} := (A_{1,1}, A_{1,2}, A_{2,1}, A_{2,2})$.

By definition of the ecological pressure we have

$$
\rho(t + \delta t) - \rho(t) = -\int_t^{t+\delta t} P(t')dt' \tag{1}
$$

Writing $P(t')$ as a Taylor expansion around t and taking the integral we find

$$
\rho(t + \delta t) - \rho(t) = -P(t)\delta t - \dot{P}(t)\frac{(\delta t)^2}{2} + \cdots \tag{2}
$$

On the other hand, ecological pressure alters through time due to changes in the state variables, which are the ρ and the κ terms, i.e. $P(\rho(t), \kappa(t))$. Thus,

$$
\rho(t + \delta t) - \rho(t) = -P(\rho(t), \kappa(t))\delta t - \frac{\partial P}{\partial \rho} \cdot \dot{\rho}\frac{(\delta t)^2}{2} - \frac{\partial P}{\partial \kappa} \cdot \dot{\kappa}\frac{(\delta t)^2}{2} \cdots \tag{3}
$$

For $\delta t << 1$ we have

$$\delta\rho(t) \approx -P(\rho(t), \kappa(t))\delta t \tag{4}$$

As a thought experiment, consider planting a cohort of individuals at time t with a random pattern. Over short periods of time P depends only on the individuals that are alive at time t (the individuals that were planted), meaning that only these individuals contribute to the immediate change in density. Equation (4) also indicates that the next generation of individuals doesn't play an active role in the production of new offspring in the short-term following the initial planting. As has been demonstrated previously, the triadic mechanism cannot be explained with the relation (4), meaning it is expressed over longer time periods. It also means that the activity of the initial population is not sufficient to generate the mechanism. In other words, the triadic mechanism is expressed only once the first offspring take an active role in the generation of new individuals. From this we can infer that the forces of this mechanism are first felt by the grandchildren of the original cohort.
